# Thyroid Hormones and Moderate Exposure to Perchlorate during Pregnancy in Women in Southern California

**DOI:** 10.1289/ehp.1409614

**Published:** 2015-10-20

**Authors:** Craig Steinmaus, Michelle Pearl, Martin Kharrazi, Benjamin C. Blount, Mark D. Miller, Elizabeth N. Pearce, Liza Valentin-Blasini, Gerald DeLorenze, Andrew N. Hoofnagle, Jane Liaw

**Affiliations:** 1 Office of Environmental Health Hazard Assessment, California Environmental Protection Agency, Oakland, California, USA; 2 School of Public Health, University of California, Berkeley, Berkeley, California, USA; 3 Sequoia Foundation, La Jolla, California, USA; 4 Environmental Health Investigations Branch, California Department of Public Health, Richmond, California, USA; 5 Division of Laboratory Sciences, National Center for Environmental Health, Centers for Disease Control and Prevention, Atlanta, Georgia, USA; 6 Pediatric Environmental Health Specialty Unit, University of California, San Francisco, San Francisco, California, USA; 7 Section of Endocrinology, Diabetes, and Nutrition, Boston University School of Medicine, Boston, Massachusetts, USA; 8 Division of Research, Kaiser Permanente Northern California, Oakland, California, USA; 9 Department of Lab Medicine, University of Washington, Seattle, Washington, USA

## Abstract

**Background:**

Findings from national surveys suggest that everyone in the United States is exposed to perchlorate. At high doses, perchlorate, thiocyanate, and nitrate inhibit iodide uptake into the thyroid and decrease thyroid hormone production. Small changes in thyroid hormones during pregnancy, including changes within normal reference ranges, have been linked to cognitive function declines in the offspring.

**Objectives:**

We evaluated the potential effects of low environmental exposures to perchlorate on thyroid function.

**Methods:**

Serum thyroid hormones and anti-thyroid antibodies and urinary perchlorate, thiocyanate, nitrate, and iodide concentrations were measured in 1,880 pregnant women from San Diego County, California, during 2000–2003, a period when much of the area’s water supply was contaminated from an industrial plant with perchlorate at levels near the 2007 California regulatory standard of 6 μg/L. Linear regression was used to evaluate associations between urinary perchlorate and serum thyroid hormone concentrations in models adjusted for urinary creatinine and thiocyanate, maternal age and education, ethnicity, and gestational age at serum collection.

**Results:**

The median urinary perchlorate concentration was 6.5 μg/L, about two times higher than in the general U.S. population. Adjusted associations were identified between increasing log10 perchlorate and decreasing total thyroxine (T4) [regression coefficient (β) = –0.70; 95% CI: –1.06, –0.34], decreasing free thyroxine (fT4) (β = –0.053; 95% CI: –0.092, –0.013), and increasing log10 thyroid-stimulating hormone (β = 0.071; 95% CI: 0.008, 0.133).

**Conclusions:**

These results suggest that environmental perchlorate exposures may affect thyroid hormone production during pregnancy. This could have implications for public health given widespread perchlorate exposure and the importance of thyroid hormone in fetal neurodevelopment.

**Citation:**

Steinmaus C, Pearl M, Kharrazi M, Blount BC, Miller MD, Pearce EN, Valentin-Blasini L, DeLorenze G, Hoofnagle AN, Liaw J. 2016. Thyroid hormones and moderate exposure to perchlorate during pregnancy in women in Southern California. Environ Health Perspect 124:861–867; http://dx.doi.org/10.1289/ehp.1409614

## Introduction

Perchlorate has been used industrially as an oxidizer in rocket propellant, road flares, and explosives. Human exposure can occur through food or water from natural or industrial sources. At medicinal levels, perchlorate blocks iodide uptake into the thyroid (Wyngaarden et al. 1953). Because iodide is a component of thyroid hormone, this can lead to decreased production of this hormone. Thyroid hormone is critical for neurodevelopment, and studies have shown that even small changes in maternal levels during pregnancy can be associated with 5- to 10-point decrements in IQ and other cognitive declines (Haddow et al. 1999; Pop et al. 2003). Thiocyanate and nitrate also block thyroid iodide uptake and may have additive impacts with perchlorate (Tonacchera et al. 2004). Thiocyanate is commonly found in vegetables and other foods and is a metabolite of cyanide in tobacco smoke. Nitrate is commonly found in vegetables, cured meats, and contaminated water.

In analyses of the U.S. National Health and Nutrition Examination Survey (NHANES), detectable urinary levels of perchlorate (> 0.05 μg/L) were found in all 10,449 participants assessed (median, 3.6 μg/L; 95th percentile, 14 μg/L in 2001–2002) (Blount et al. 2007; Steinmaus et al. 2013). Further analysis of NHANES identified associations between increasing urinary perchlorate and decreasing serum total thyroxine (T4), with the strongest association found in women with low urinary iodine and high urinary thiocyanate (Blount et al. 2006; Steinmaus et al. 2013). Clear associations were not seen in pregnant women, although the sample sizes were small (e.g., 141 women; Suh et al. 2014).

We evaluated perchlorate–thyroid hormone associations using urine and serum samples collected as part of Project Baby’s Breath (PBB), a study of tobacco smoke exposure in pregnant women in San Diego County, California, during the years 2000–2003. During this period, the largest source of drinking water in this county, the Colorado River, was contaminated with perchlorate from a perchlorate manufacturing plant located upriver (U.S. EPA 2005). Perchlorate concentrations in the Colorado River during this time were approximately 4–8 μg/L, which is near the current California regulatory standard of 6 μg/L. U.S. survey data from 2005 through 2006 suggest that median tap water perchlorate concentrations are around 1 μg/L (Blount et al. 2010).

## Methods

### Study Population and Data Collection

The participants were a convenience sample of pregnant women obtaining health care in San Diego County, who delivered from November 2000 to March 2003, and were in PBB. Participants were recruited at several collection periods and sites, including 37 community clinics and obstetrical care providers, the statewide prenatal screening program, and 15 birthing facilities throughout the county. Urine samples assayed for the present analysis were left over from spot urine samples collected for pregnancy tests at a median of 7 weeks gestation. After pregnancy testing, clinic staff transferred the remaining urine into 5-mL Corning cryovials that were refrigerated and transferred within 1 day to a central laboratory for storage at –20°C. Perchlorate is stable for many months at room temperature (Stetson et al. 2006). Serum samples assayed for the present analysis were left over from samples collected by obstetrical care providers at approximately 15–20 weeks gestation from women who participated in the California Prenatal Screening (PNS) Program (Cunningham and Tompkinison 1999). After collection in 4-mL serum separator tubes, specimens were spun down and tested for chromosomal abnormalities and neural tube defects, with a median time between collection and PNS testing of 3 days. After PNS testing and 1–2 days of refrigeration, remaining serum from PBB study participants was transferred to 4-mL Corning cryovials and stored at –20°C. Samples not assayed within 7 days of collection were excluded from the PNS and not available for PBB. Männistö et al. (2007) reported that free thyroxine (fT4) or thyroid-stimulating hormone (TSH) were relatively stable for 6 days at 4°C and up to 23 years at –25°C. Information on mother’s age, highest education, prenatal weight, ethnicity, payment method (e.g., private insurance vs. Medi-Cal), and ethnicity was collected from birth records. Urine and serum samples, PNS program data, and birth records were linked using probabilistic matching software. PNS to birth match rates using this method are generally 93% (Kharrazi et al. 2012). Informed consent was obtained for collection of leftover urine and cord blood specimens for the PBB study and for future testing of stored specimens for environmental contaminants. PNS program participants signed a consent/refusal form and privacy notification regarding research use of their specimen. The PBB study and the study presented here were approved by the State of California Committee for the Protection of Human Subjects. The study presented here was also approved by the University of California, Berkeley, Committee for the Protection of Human Subjects.

### Laboratory Measurements

Urine is the most common matrix for evaluating perchlorate exposure, because all ingested perchlorate is excreted unchanged in the urine (Blount and Valentin-Blasini 2007). Urine samples were shipped overnight to the Center for Disease Control and Prevention (CDC) on dry ice and analyzed by the CDC’s Perchlorate Biomonitoring Laboratory for perchlorate (detection limit, 0.05 μg/L), thiocyanate (20 μg/L), nitrate (700 μg/L), and iodide (0.5 μg/L) using ion chromatography tandem mass spectrometry (Valentin-Blasini et al. 2007). Results met the division’s quality control criteria for accuracy and precision (Caudill et al. 2008). After overnight shipping on dry ice, serum samples were measured for total T4, fT4, TSH, and thyroperoxidase (TPO) and thyroglobulin (TG) antibody concentrations at University of Washington, Seattle, using a Beckman automated immunoassay chemiluminescence platform (Beckman Coulter) and microparticle enzyme immunoassay (Abbott Laboratories). Quality control measures included two-level quantitative controls for each assay on every reagent run, monitoring run integrity using Levey–Jennings charts, and participation in proficiency surveys by the College of American Pathologists. Manufacturers’ values for TPO and TG antibody positivity are > 9 and > 4 IU/mL, respectively.

### Statistical Analysis

Statistical analyses were done using SAS version 9.1 (SAS Institute Inc.), and all *p*-values are two-sided. Univariate analyses and data plots were performed to examine distributions and identify outliers. Eight participants with very high T4 (> 20 μg/dL, similar to the cutoff in a previous study) or TSH values (> 10 μg/dL, a level above which thyroid hormone initiation is recommended) (Blount et al. 2006; Garber et al. 2012) were excluded from the present analysis, although this had little impact on results (data not shown). Excluding participants with urine concentrations of perchlorate, thiocyanate, nitrate, and iodide above the 99th percentile also had little impact on associations with the markers of thyroid function (data not shown). Perchlorate, thiocyanate, creatinine, and TSH were log_10_ transformed to create normal distributions. Linear regression was used to evaluate associations between perchlorate and each thyroid hormone, with both perchlorate and the thyroid hormone as continuous variables. Variables available from vital and prenatal records that were evaluated for inclusion to the models included maternal age and prenatal weight (continuous), education (< 9, 9–11, 12, or > 12th grade), ethnicity (Hispanic vs. non-Hispanic), multiple births [one vs. more than one (e.g., twins)], payment method (private vs. public), previous births (number of previous births), and gestational age at serum collection (continuous). Urinary creatinine concentrations, maternal age and education, ethnicity, and gestational age at serum collection were selected for the final models *a priori*. Of the remaining variables assessed for model inclusion [urinary thiocyanate, nitrate, iodide, birthplace (United States vs. other), antithyroid antibodies (positive vs. negative), and prenatal weight], only urinary thiocyanate changed regression coefficients by > 10% and was also entered into the final models. Directed acyclic graphs were used to confirm that the variables included were appropriate for adjustment (data not shown).

Creatinine-adjusted residuals for perchlorate and the other analytes were calculated using the methods described elsewhere (Willet and Stampfer 1998). Urinary creatinine is commonly used to adjust for urine dilution but may be influenced by other factors including muscle mass, illness, exercise, and diet (Barr et al. 2005). This can create a situation in which some people’s creatinine-adjusted perchlorate exposure ends up being determined more by their creatinine concentration (and the other factors related to creatinine) than by their true perchlorate exposure. This could lead to substantial misclassification, especially in those with very high or very low creatinine values (Steinmaus et al. 2009). For this reason, participants with urine creatinine concentrations > 90th percentile (233 mg/dL) and < 10th percentile (41 mg/dL) were excluded in our main analyses.

Perchlorate–thyroid hormone associations were also estimated after stratifying by urinary iodide categories of < 100, 100–300, and > 300 μg/L, corresponding to values used by the World Health Organization to define iodine deficiency, normal values, and elevated iodine levels, respectively, in non-pregnant populations ≥ 6 years old (WHO 2007). In addition, we performed analyses stratified by thiocyanate and nitrate concentrations categorized into three groups according to the lowest quartile, the 25–75th percentile, and the highest quartile, respectively, similar to categories used in a previous analysis of NHANES data (Steinmaus et al. 2007). Interactions were assessed using product terms (log_10_ perchlorate × log_10_ thiocyanate and log_10_ perchlorate × log_10_ nitrate), using a two-sided *p*-value < 0.05 for each product term as indicating statistically significant interaction. Trend test *p*-values to assess linear dose–response relations were derived by using proc GLM to model an ordinal variable representing quartiles of exposure to perchlorate, iodide, thiocyanate, or nitrate, with participants assigned the mean value of their respective quartile. In all analyses, two-sided *p*-values of < 0.05 were used to indicate statistical significance.

## Results

Urinary perchlorate and serum thyroid hormone concentrations were available on 1,880 women. The median concentrations of perchlorate and iodide were 6.50 and 154.5 μg/L, respectively (Table 1). This perchlorate concentration is more than two times higher than that reported in women in NHANES 2001–2002 (median, 3.0 μg/L) (Blount et al. 2007). The median concentrations of total T4, fT4, and TSH were 12.26 μg/dL (range, 0.56–19.99), 0.85 ng/dL (range, 0.11–5.44), and 1.20 μIU/mL (range, 0.003–8.39), respectively.

**Table 1:** Distributions of maternal urinary analytes and serum thyroid hormones.

Analyte	*n*	Mean ± SD	Minimum	Percentile			Maximum
				25th	50th	75th	
Urine perchlorate (μg/L)	1,880	8.49 ± 9.86	0.23	3.95	6.50	9.96	177.00
Urine thiocyanate (μg/L)	1,880	1,353 ± 1,553	20	459	898	1,590	16,200
Urine nitrate (mg/L)	1,876	66.5 ± 57.0	0.7	32.9	55.5	84.3	796.0
Urine iodide (μg/L)	1,818	214.9 ± 247.3	0.8	77.0	154.5	270.0	3,000
Urine creatinine (mg/dL)	1,878	131.5± 75.7	7.9	73.3	120.9	175.0	472.5
Serum total T4 (μg/dL)^*b*^	1,880	12.31 ± 1.89	0.56	11.21	12.26	13.40	19.99
Serum TSH (μIU/mL)^*b*^	1,879	1.36 ± 0.86	0.003	0.80	1.20	1.69	8.39
Serum fT4 (ng/dL)^*b*^	1,880	0.86 ± 0.19	0.11	0.77	0.85	0.93	5.44
TG antibody (IU/mL)	1,879	3.11 ± 24.13	0.00	0.00	0.00	0.60	565.90
TPO antibody (IU/mL)	1,876	12.90 ± 59.73	0.10	0.50	0.80	1.50	704.90
Urine collection (week)	1,878	8.6 ± 5.2	0.1	5.7	7.1	10.0	42.9
Serum collection (week)	1,880	17.1 ± 1.5	9.1	16.1	17.0	18.0	26.0
Urine-serum difference (weeks)^*a*^	1,878	9.2 ± 3.8	0.0	7.0	9.9	11.6	26.7
Abbreviations: fT4, free thyroxine; T4, total thyroxine; TG, thyroglobulin; TPO, thyroperoxidase; TSH, thyroid-stimulating hormone. ^***a***^Absolute difference between urine and serum collection times. Seventy-two participants (3.8%) had their urine samples collected after their serum samples. The mean urine-serum difference considering these negative values was 8.4 ± 5.2 weeks; range, –26.7–19.3 weeks. ^***b***^Reference ranges from the University of Washington for all ages and both sexes are 4.8–10.8 μg/dL for total T4; 0.6–1.2 ng/dL for fT4, and 0.4–5.0 μIU/mL for TSH. The American Thyroid Association upper reference range for TSH in the second trimester of pregnancy is 3.0 μIU/mL (Garber et al. 2012).							

The median age and education level achieved was 25 years [interquartile range (IQR), 21–29] and 12 years (IQR, 9–12), respectively, and 69.3% of participants were Hispanic and 47.3% were born in Mexico (Table 2). Increasing maternal and gestational age at serum collection were associated with decreasing total T4 and fT4 concentrations but not with TSH. Mean total T4 levels were higher in Hispanic women than in women in other racial/ethnic groups, though the difference was only statistically significant (*p* < 0.05) between Hispanic women and white and black women. Women born in Mexico had significantly higher total T4 levels than women born in the United States. Urine concentrations of perchlorate, thiocyanate, nitrate, and iodide were positively correlated with each other (Spearman correlation coefficients between 0.22 and 0.43), although correlations were reduced after creatinine adjustment (see Table S1). For example, correlations between perchlorate and nitrate before and after creatinine adjustment were 0.41 (*p* < 0.001) and 0.26 (*p* < 0.001), respectively.

**Table 2:** Maternal thyroid hormone concentrations stratified by various demographic factors.

Category	*n* (%)	T4 (μg/dL)			fT4 (ng/dL)			TSH (μIU/mL)^*a*^		
		Median	Mean ± SD	*p*-Value^*b*^	Median	Mean ± SD	*p*-Value^*b*^	Median	Mean ± SD	*p*-Value^*b*^
Mother’s age (years)
< 21	435 (23.1)	12.43	12.50 ± 1.87		0.87	0.87 ± 0.14		1.26	1.36 ± 0.77
21–25	563 (29.9)	12.36	12.45 ± 1.91		0.86	0.87 ± 0.23		1.16	1.35 ± 0.88
26–30	414 (22.0)	12.26	12.38 ± 1.88		0.84	0.85 ± 0.24		1.16	1.36 ± 0.98
> 30	468 (24.9)	11.91	11.90 ± 1.83		0.83	0.83 ± 0.13		1.23	1.38 ± 0.80
*R*			–0.12	< 0.001		–0.14	< 0.001		0.01	0.78
Mother’s education (highest grade)
< 9	509 (27.6)	12.42	12.57 ± 1.96		0.84	0.86 ± 0.30		1.12	1.33 ± 0.90
9–11	308 (16.7)	12.31	12.35 ± 2.08		0.85	0.86 ± 0.15		1.22	1.41 ± 0.87
12	568 (30.8)	12.23	12.22 ± 1.81		0.85	0.86 ± 0.13		1.21	1.32 ± 0.82
> 12	460 (24.9)	12.05	12.07 ± 1.73		0.85	0.85 ± 0.12		1.26	1.44 ± 0.86
*R*			–0.10	< 0.001		0.02	0.31		0.07	0.003
Mother’s weight (lbs)
< 121	469 (24.9)	12.38	12.49 ± 1.77		0.87	0.89 ± 0.25		1.15	1.32 ± 0.85
122–145	471 (25.1)	12.10	12.09 ± 1.80		0.85	0.86 ± 0.12		1.17	1.31 ± 0.82
146–171	466 (24.8)	12.31	12.39 ± 1.81		0.85	0.86 ± 0.23		1.23	1.38 ± 0.87
> 171	474 (25.2)	12.25	12.27 ± 2.13		0.83	0.83 ± 0.14		1.27	1.44 ± 0.90
*R*			–0.01	0.54		–0.14	< 0.001		0.06	0.01
Serum collection (weeks gestation)
< 16	467 (24.8)	12.48	12.56 ± 1.75		0.88	0.89 ± 0.14		1.15	1.29 ± 0.86
17	512 (27.2)	12.20	12.32 ± 1.84		0.85	0.86 ± 0.21		1.25	1.37 ± 0.77
18	448 (23.8)	12.15	12.16 ± 1.82		0.82	0.84 ± 0.12		1.16	1.36 ± 0.87
> 18	453 (24.1)	12.25	12.19 ± 2.11		0.83	0.84 ± 0.25		1.24	1.43 ± 0.94
*R*			–0.07	0.004		–0.19	< 0.001		0.04	0.08
TG (IU/mL)
≤ 4	1,719 (91.4)	12.22	12.25 ± 1.83		0.85	0.86 ± 0.20		1.20	1.36 ± 0.82
> 4	161 (8.6)	12.97	12.97 ± 2.30	< 0.001	0.83	0.84 ± 0.13	0.05	1.17	1.43 ± 1.25	0.16
TPO (IU/mL)
≤ 9	1,715 (91.2)	12.26	12.33 ± 1.87		0.85	0.86 ± 0.20		1.16	1.28 ± 0.71	
> 9	165 (8.8)	12.23	12.10 ± 2.07	0.47	0.82	0.82 ± 0.13	0.006	1.65	2.21 ± 1.54	< 0.001
Ethnicity
Hispanic	1,303 (69.3)	12.38	12.47 ± 1.90	Ref	0.85	0.86 ± 0.21	Ref	1.17	1.34 ± 0.86	Ref
White	391 (20.8)	11.88	11.87 ± 1.72	< 0.001	0.84	0.84 ± 0.12	0.17	1.31	1.52 ± 0.91	< 0.001
Asian	72 (3.8)	12.30	12.17 ± 2.27	0.54	0.84	0.83 ± 0.14	0.48	1.20	1.23 ± 0.66	0.53
Black	24 (1.3)	11.92	11.37 ± 1.84	0.01	0.90	0.90 ± 0.13	0.10	1.19	1.44 ± 0.83	0.55
Other	90 (4.8)	12.01	12.32 ± 1.74	0.16	0.87	0.90 ± 0.19	0.06	0.94	1.04 ± 0.69	< 0.001
Mother’s birthplace
USA	854 (45.4)	12.10	12.16 ± 1.81	Ref	0.85	0.85 ± 0.13	Ref	1.25	1.42 ± 0.86	Ref
Mexico	889 (47.3)	12.38	12.45 ± 1.97	0.001	0.84	0.86 ± 0.24	0.65	1.16	1.32 ± 0.87	0.002
Other	137 (7.3)	12.28	12.35 ± 1.79	0.20	0.88	0.90 ± 0.14	< 0.001	1.13	1.24 ± 0.73	0.02
Abbreviations: fT4, free thyroxine; *R*, Spearman correlation coefficient; Ref, reference category; T4, total thyroxine; TG, thyroglobulin antibody; TPO, thyroperoxidase antibody; TSH, thyroid stimulating hormone. ^***a***^One subject was missing a serum TSH value. ^***b***^When the variables are continuous, *p*-values are provided for the Spearman correlation coefficient between the two continuous variables. When variables are ordinal, *p*-values are provided comparing the mean thyroid hormone value in each group to a reference group.										

Among women with creatinine concentrations between the 10th and 90th percentiles (1,476 of the 1,880 women enrolled in the PBB), regression coefficients for associations between log_10_ perchlorate and total T4, fT4, and log_10_ TSH were –0.70 [95% confidence interval (CI): –1.06, –0.34], –0.053 (95% CI: –0.092, –0.013), and 0.071 (95% CI: 0.008, 0.133), respectively, after adjustment for urine creatinine, urine thiocyanate, maternal age, maternal education, ethnicity, and gestational age at serum collection (Table 3). Thus, each 10-fold increase in perchlorate was associated with a 0.70-μg/L decrease in T4 and a 0.053-μg/L decrease in fT4, and each 1% increase in perchlorate was associated with a 0.071% increase in TSH. Additional adjustment for maternal weight, payment method, time between sample urine and serum sample collection, number of previous births, or nitrate and iodine concentrations had little impact on associations between log_10_ perchlorate and thyroid hormones (data not shown). Results were similar when we restricted our subjects to a narrower age range. For example, the log_10_ perchlorate–total T4 regression coefficient (in the creatinine limited set) was –0.70 (95% CI: –1.06, –0.34) for all ages and –0.78 (95% CI: –1.22, –0.34) for women ages 20–30. Associations between log_10_ perchlorate and total T4 were stronger among women in the highest quartile of thiocyanate (–1.28; 95% CI: –2.04, –0.51) compared with women in the lowest quartile (–0.41; 95% CI: –1.05, 0.24), among women in the highest versus lowest quartile of nitrate intake (–1.04; 95% CI: –1.70, –0.38 compared with –0.20; 95% CI: –0.88, 0.48), among women who were anti-thyroid antibody positive versus negative (–1.55; 95% CI: –2.75, –0.34 compared with –0.52; 95% CI: –0.89, –0.15), and among women with elevated urine iodide concentrations (–1.77; 95% CI: –2.73, –0.81) compared with women classified as having normal urine iodide (–0.44; 95% CI: –0.95, 0.08), although patterns of associations with fT4 and TSH according to population subgroup were not consistent with patterns of associations with total T4 according to population subgroup. Interactions between log_10_ perchlorate and log_10_ nitrate or log_10_ thiocyanate modeled as continuous variables were not statistically significant (*p* > 0.05) (data not shown).

**Table 3:** Regression coefficients between maternal log_10_ urine perchlorate concentrations and serum thyroid hormone concentrations.*^a^*

Group	*n*	T4 [β^*b*^ (95% CI)]	fT4 [β^*b*^ (95% CI)]	Log_10_TSH [β^*b*^ (95% CI)]
All subjects	1,476	–0.70 (–1.06, –0.34)	–0.053 (–0.092, –0.013)	0.071 (0.008, 0.133)
Iodide
< 100 μg/L	452	–0.57 (–1.20, 0.07)	–0.022 (–0.100, 0.056)	0.034 (–0.081, 0.148)
100–300 μg/L	698	–0.44 (–0.95, 0.08)	–0.031 (–0.067, 0.005)	0.094 (0.002, 0.189)
> 300 μg/L	326	–1.77 (–2.73, –0.81)	–0.172 (–0.305, –0.039)	0.089 (–0.058, 0.236)
Thiocyanate
< 519 μg/L	360	–0.41 (–1.05, 0.24)	–0.034 (–0.074, 0.006)	0.039 (–0.092, 0.170)
519–1,620 μg/L	741	–0.74 (–1.27, –0.21)	–0.082 (–0.154, –0.010)	0.086 (0.005, 0.166)
> 1,620 μg/L	375	–1.28 (–2.04, –0.51)	–0.041 (–0.098, 0.016)	0.115 (–0.021, 0.252)
Nitrate
< 36.3 mg/L	366	–0.20 (–0.88, 0.48)	–0.040 (–0.133, 0.054)	0.053 (–0.071, 0.178)
36.3–81.9 mg/L	737	–0.72 (–1.32, –0.12)	–0.062 (–0.130, 0.006)	0.093 (–0.014, 0.200)
> 81.9 mg/L	369	–1.04 (–1.70, –0.38)	–0.057 (–0.101, –0.013)	0.099 (0.000, 0.199)
Ethnicity
Hispanic	1,035	–0.78 (–1.21, –0.36)	–0.073 (–1.124, –0.023)	0.037 (–0.032, 0.106)
Non-Hispanic	441	–0.59 (–1.27, 0.09)	–0.002 (–0.054, 0.050)	0.184 (0.052, 0.317)
Sample collection^*c*^
≤ 8 weeks apart	470	–1.05 (–1.73, –0.38)	–0.052 (–0.136, 0.033)	0.001 (–0.121, 0.122)
> 8 weeks apart	1,006	–0.55 (–0.98, –0.13)	–0.050 (–0.092, –0.008)	0.107 (0.036, 0.178)
Anti-thyroid antibodies
Positive^*d*^	203	–1.55 (–2.75, –0.34)	–0.093 (–0.159, –0.027)	0.153 (–0.060, 0.365)
Negative	1,273	–0.52 (–0.89, –0.15)	–0.047 (–0.091, –0.002)	0.060 (–0.004, 0.123)
Abbreviations: β, regression coefficient; fT4, free thyroxine; T4, total thyroxine; TSH, thyroid-stimulating hormone. ^***a***^Only includes participants with urinary creatinine concentrations between the 10th and 90th percentiles (i.e., 41–233 mg/dL). ^***b***^Adjusted for urinary creatinine, maternal age, maternal education, ethnicity (Hispanic vs. non-Hispanic), gestational age at serum collection, and urinary thiocyanate. ^***c***^Weeks between urine and serum sample collection. ^***d***^Includes participants with thyroperoxidase or thyroglobulin antibody concentrations above 9 and 4 IU/mL, respectively.				

Only small differences were seen between adjusted and unadjusted results in most analyses (data not shown). For log perchlorate and fT4, the unadjusted regression coefficient, creatinine-only adjusted regression coefficient, and fully adjusted regression coefficient (creatinine, maternal age, maternal education, ethnicity, gestational age at serum collection, and urinary thiocyanate) in the creatinine restricted data set were –0.045 (95% CI: –0.082, –0.010), –0.055 (95% CI: –0.093, –0.017), and –0.053 (95% CI: –0.092, –0.013), respectively. Statistically significant trends in decreasing total T4 values and decreasing fT4 values with increasing perchlorate quartiles were seen in categorical analyses of perchlorate quartiles (see Table S2). Including participants with urine creatinine concentrations < 10th percentile and > 90th percentile generally resulted in perchlorate–thyroid hormone regression coefficients somewhat less in magnitude (data not shown). For example, the adjusted regression coefficients between log_10_ perchlorate and total T4, fT4, and TSH excluding these subjects were –0.70 (95% CI: –1.06, –0.34), –0.053 (95% CI: –0.092, –0.013), and 0.071 (95% CI: 0.008, 0.133), respectively, and the corresponding regression coefficients including these subjects were –0.54 (95% CI: –0.85, *–*0.25), –0.047 (95% CI: –0.079, –0.016), and 0.049 (95% CI: –0.004, 0.104).

## Discussion

These findings provide evidence that environmental perchlorate exposure is associated with decreased production of thyroid hormone in mid-pregnancy. In our main analyses, increasing perchlorate concentrations were associated with decreasing T4, decreasing fT4, and increasing TSH. Although the issue of multiple comparisons may be a concern, the direction of each of these relationships is consistent with the well-established mechanism of perchlorate. In addition, negative associations between perchlorate and total T4 were stronger in women with urinary nitrate and thiocyanate concentrations in the highest versus lowest quartiles. These results are consistent with *in vitro* data showing that perchlorate, nitrate, and thiocyanate can have additive effects on inhibiting iodine uptake into thyroid cells (Tonacchera et al. 2004). Anti-thyroid antibodies have been associated with altered thyroid function (Hollowell et al. 2002; Pearce et al. 2008), and negative associations between perchlorate and fT4 were stronger in our study among women who were antibody positive versus negative though formal tests of differences between the two groups were not done. Overall, these results suggest that certain groups, including those exposed to other iodine-inhibiting agents and those who are anti-thyroid antibody positive, may be particularly susceptible to the adverse impacts of perchlorate.

Several previous studies have not identified clear associations between perchlorate and thyroid hormones (Pearce et al. 2010), but many of these involved smaller sample sizes (Pearce et al. 2011, 2012; Suh et al. 2014), limited exposure periods (Greer et al. 2002), or involved only healthy adults (Braverman et al. 2006). Our results are consistent with several other studies that have examined potentially susceptible groups, including infants, those exposed to other thyroid inhibitors, and those with low iodine intakes (Brechner et al. 2000; Cao et al. 2010; Charatcharoenwitthaya et al. 2014; Mendez and Eftim 2012; Steinmaus et al. 2010).

In contrast to some previous results from NHANES (Blount et al. 2006), associations between perchlorate and thyroid hormones were similar between women with low urine iodide concentrations and women with normal iodide concentrations. One reason for this could be the overall iodine sufficiency in this population. The median urinary iodide level of 154.5 μg/L is above the level of 150 μg/L used by the WHO to define iodine sufficiency in a pregnant population (WHO 2007). Another reason could be the fairly long time between urine iodine and serum thyroid hormone sample collection (about 9 weeks). Changes in diet (and dietary iodine intake) or changes in the use of prenatal vitamins containing iodine during this time could have led to changes in iodine intake in some participants. If these changes occurred, our measure of iodine status may not have reflected true long-term iodine status or iodine status at the time thyroid hormones were measured. Although the actual bias this may have caused in this study is unknown, this type of measurement error would have most likely caused a nondifferential misclassification and most likely biased any true impacts of low iodide status toward the null.

We did identify a greater perchlorate–fT4 association in women with very high urinary iodide concentrations (i.e., > 300 μg/L). In most individuals, very high iodine intakes transiently and paradoxically inhibit thyroid hormone production, termed the acute Wolff–Chaikoff effect (Wolff and Chaikoff 1948). Normally, this is only temporary, and after a short disruption there is an “escape” and thyroid function returns to normal after a few days, even if high iodine exposure continues (Eng et al. 1999). However, several studies have reported increased rates of thyroid autoimmunity and hypothyroidism in areas where people have chronically high iodine intakes, from diet (e.g., seaweed consumption) or drinking water with naturally high iodine concentrations. This would suggest that chronic excessive high iodine intakes can lead to long-term hypothyroidism in susceptible individuals (Konno et al. 1994; Li et al. 1987; Pedersen et al. 2007; Tajiri et al. 1986; Teng et al. 2006). Associations between very high urinary iodide concentrations and decreased thyroid hormone production have also been seen in NHANES (Cushing et al. 2011; Vanderver et al. 2007). Overall, these studies, combined with our findings, suggest that excessive iodine intakes could lead to altered thyroid function and an enhanced susceptibility to perchlorate.

The results of this study are based on single assessments of urinary concentrations of perchlorate, iodine, thiocyanate, nitrate, and serum thyroid hormones separated by an average of 9 weeks. All of these can vary throughout the day and from day to day. The direction of any bias caused by this variability is unpredictable; however, these variations would most likely reduce statistical power or most likely bias any true associations to the null (Roy 1994). The half-life of perchlorate excretion is fairly short (e.g., 48–72 hr) (Greer et al. 2002; Lamm et al. 1999; Selivanova and Arefaeva 1986). A study in New York City children following urinary perchlorate levels over a 6-month period has shown that a single measurement can be used to accurately classify participants into low, medium, and high long-term exposure groups, though it is unknown how generalizable these results are to our study population (Mervish et al. 2011). Maternal thyroid hormone levels change rapidly during the first part of gestation due largely to the effects of human chorionic gonadotropin (hCG), which is a stimulator of the thyroidal TSH receptor and thus drives increased thyroid hormone production in the first trimester (Krassas et al. 2010). Levels of hCG peak at about 10 weeks gestation then decline. Thus, single measurements of thyroid function early in gestation might not reflect true long-term levels. In addition, pregnancy-related changes in serum proteins can impact fT4 immunoassays (Lee et al. 2009). Importantly, the serum samples in this study were collected and analyzed similarly in all participants regardless of perchlorate levels. Therefore, any misclassification of these variables is likely to be nondifferential and would not likely cause the positive associations identified here (Roy 1994).

Urinary creatinine concentration is influenced by many factors including age, race, weight, health, and other factors, and creatinine adjustments can potentially introduce significant misclassification of other urinary metabolites including perchlorate (Barr et al. 2005; Steinmaus et al. 2009). For example, an analysis of NHANES data showed that creatinine adjustment of urinary iodine caused an extensive redistribution of participants into different iodine categories, and that much of this redistribution was likely related to age rather than urine dilution (Haddow et al. 2007). Misclassification such as this would most likely be greater in people with extreme values of creatinine and removing these extreme values was done in our study in an effort to help reduce the numbers of subjects misclassified as a result of this type of inappropriate creatinine adjustment.

Our analyses incorporated several factors that influence thyroid hormone levels on a population basis, including maternal age, gestational age, and ethnicity. We used maternal education or payment method as indicators of socioeconomic status (SES). Incorporating other SES-related variables such as household income may have allowed us to develop a more comprehensive SES assessment. We did not have information on some of the other factors that may influence thyroid hormone levels, including certain genetic conditions, some medications, or the presence of thyroid disease. Some of these factors, including the use of most medications during pregnancy or genetic abnormalities, are likely too rare to cause major confounding. Some studies have linked diet to thyroid hormone concentrations, but much of this involves dietary thiocyanate, nitrate, or iodine (Bourdoux et al. 1978; Ward et al. 2010), variables we assessed in this study. Overall, we did not see evidence of major confounding in this study, although as with almost any epidemiologic study, unknown confounding cannot be completely excluded.

Based on the log perchlorate–fT4 regression coefficient of –0.053, we estimate that people with perchlorate exposures at the upper 95th percentile value in our study (20.1 μg/L) would have mean fT4 values that are about 12% lower than people with perchlorate exposures at the lowest value measured in our study (0.23 μg/L). Although relatively small, this level of impact could have important implications for those who have borderline low fT4 concentrations for reasons other than perchlorate. In these people, any additional impact of perchlorate could push their fT4 concentrations to a level at which they start developing adverse hypothyroid symptoms. In addition, several studies have shown that even small changes in fT4 and TSH during pregnancy, including those within normal reference ranges, as well as mild maternal iodine deficiency, may be associated with 5–10% decreases in IQ and other important cognitive declines in the offspring (Bath et al. 2013; Haddow et al. 1999; Henrichs et al. 2010; Hynes et al. 2013; Li et al. 2010; Pop et al. 2003; Vermiglio et al. 2004). In a recent historical cohort study of 21,846 women in Cardiff, United Kingdom, and Turin, Italy, who were pregnant from 2002 to 2006, urinary perchlorate levels and subsequent childhood IQ were examined in 487 mother–child pairs in mothers who were hypothyroid/hypothyroxinemic during pregnancy. This study reported that although associations were not seen with maternal thyroid function, mothers with urine perchlorate concentrations in the highest 10% were at increased risk for having children with IQ scores in the lowest decile at age three (Taylor et al. 2014). Overall, these studies highlight the potential importance of both perchlorate exposure and small changes in thyroid function during pregnancy.

Given that most people’s exposure to perchlorate is relatively low, the associations identified here, if causal, might result in small or even unnoticeable effects in most exposed individuals. However, given the widespread nature of perchlorate exposure, even small effects could have large impacts on a population basis.

## Conclusions

The public health significance of this study lies in the large number of people exposed to perchlorate, the large numbers exposed to other thyroid-active agents such as nitrate and thiocyanate, and the importance of thyroid hormone in neurodevelopment. The results of this study suggest that environmental exposures to perchlorate may affect thyroid hormone production in pregnant women.

## Supplemental Material

(253 KB) PDF
