# Supplementary material for: Thyroid Hormones and Moderate Exposure to Perchlorate during Pregnancy in Women in Southern California
Source: Environ Health Perspect. 2015 Oct 20;124(6):861–7. doi: 10.1289/ehp.1409614 (PMC4892913; doi:10.1289/ehp.1409614)
Supplement: (253 KB) PDF [file ehp.1409614.s001.acco.pdf]

Environ Health Perspect
DOI: 10.1289/ehp.1409614

Note to readers with disabilities: *EHP* strives to ensure that all journal content is accessible to all readers. However, some figures and Supplemental Material published in *EHP* articles may not conform to 508 standards due to the complexity of the information being presented. If you need assistance accessing journal content, please contact ehp508@niehs.nih.gov. Our staff will work with you to assess and meet your accessibility needs within 3 working days.

Supplemental Material

Thyroid Hormones and Moderate Exposure to Perchlorate during Pregnancy in Women in Southern California

Craig Steinmaus, Michelle Pearl, Martin Kharrazi, Benjamin C. Blount, Mark D. Miller, Elizabeth N. Pearce, Liza Valentin-Blasini, Gerald DeLorenze, Andrew N. Hoofnagle, and Jane Liaw

Table of Contents

Table S1. Correlations between maternal urine analyte concentrations and other factors
Table S2. Maternal thyroid hormone concentrations by increasing quartiles of urine perchlorate and other analyte concentrations

Table S1. Correlations between maternal urine analyte concentrations and other factors[a]

Variable		Perchlorate (µg/L)	Iodide (µg/L)	Thiocyanate (µg/L)	Nitrate (mg/L)	Creatinine (mg/dL)	Perchlorate adjusted[b]	Iodide adjusted[b]	Thiocyanate adjusted[b]	Nitrate adjusted[b]	TG antibodies (IU/mL)	TPO antibodies (IU/mL)	Urine collection time (weeks)	Serum collection time (weeks)
Perchlorate	R	1.00	0.43	0.23	0.41	0.35	0.90	0.30	0.14	0.26	-0.05	-0.04	-0.02	-0.02
(µg/L)	p		<0.001	<0.001	<0.001	<0.001	<0.001	<0.001	<0.001	<0.001	0.06	0.14	0.38	0.34
	N	1505	1454	1505	1501	1505	1505	1454	1505	1501	1504	1504	1503	1505
Iodide	R	0.43	1.00	0.27	0.31	0.41	0.28	0.90	0.18	0.12	-0.01	-0.03	0.00	-0.04
(µg/L)	p	<0.001		<0.001	<0.001	<0.001	<0.001	<0.001	<0.001	<0.001	0.79	0.23	0.96	0.16
	N	1454	1454	1454	1450	1454	1454	1454	1454	1450	1453	1453	1452	1454
Thiocyanate	R	0.23	0.27	1.00	0.22	0.26	0.12	0.17	0.96	0.13	-0.13	-0.14	-0.16	-0.01
(µg/L)	p	<0.001	<0.001		<0.001	<0.001	<0.001	<0.001	<0.001	<0.001	<0.001	<0.001	<0.001	0.83
	N	1505	1454	1505	1501	1505	1505	1454	1505	1501	1504	1504	1503	1505
Nitrate	R	0.41	0.31	0.22	1.00	0.52	0.19	0.10	0.09	0.83	-0.04	-0.07	-0.01	0.00
(mg/L)	p	<0.001	<0.001	<0.001		<0.001	<0.001	<0.001	<0.001	<0.001	0.11	0.01	0.74	0.86
	N	1501	1450	1501	1501	1501	1501	1450	1501	1501	1500	1500	1499	1501
Creatinine	R	0.35	0.41	0.26	0.52	1.00	-0.06	0.01	0.02	0.02	-0.01	-0.03	-0.04	0.00
(mg/dL)	p	<0.001	<0.001	<0.001	<0.001		0.02	0.74	0.34	0.38	0.76	0.20	0.10	0.85
	N	1505	1454	1505	1501	1505	1505	1454	1505	1501	1504	1504	1503	1505
Perchlorate	R	0.90	0.28	0.12	0.19	-0.06	1.00	0.32	0.14	0.26	-0.05	-0.03	0.00	-0.03
adjusted[b]	p	<0.001	<0.001	<0.001	<0.001	0.02		<0.001	<0.001	<0.001	0.07	0.23	0.93	0.22
	N	1505	1454	1505	1501	1505	1505	1454	1505	1501	1504	1504	1503	1505
Iodide	R	0.30	0.90	0.17	0.10	0.01	0.32	1.00	0.17	0.12	-0.01	-0.02	0.02	-0.04
adjusted[b]	p	<0.001	<0.001	<0.001	<0.001	0.74	<0.001		<0.001	<0.001	0.82	0.51	0.48	0.15
	N	1454	1454	1454	1450	1454	1454	1454	1454	1450	1453	1453	1452	1454
Thiocyanate	R	0.14	0.18	0.96	0.09	0.02	0.14	0.17	1.00	0.12	-0.14	-0.13	-0.16	-0.01
adjusted[b]	p	<0.001	<0.001	<0.001	<0.001	0.34	<0.001	<0.001		<0.001	<0.001	<0.001	<0.001	0.77
	N	1505	1454	1505	1501	1505	1505	1454	1505	1501	1504	1504	1503	1505
Nitrate	R	0.26	0.12	0.13	0.83	0.02	0.26	0.12	0.12	1.00	-0.04	-0.05	0.03	0.00
adjusted[b]	p	<0.001	<0.001	<0.001	<0.001	0.38	<0.001	<0.001	<0.001		0.13	0.06	0.32	0.90
	N	1501	1450	1501	1501	1501	1501	1450	1501	1501	1500	1500	1499	1501
TG antibodies	R	-0.05	-0.01	-0.13	-0.04	-0.01	-0.05	-0.01	-0.14	-0.04	1.00	0.29	0.00	0.00
(IU/mL)	p	0.06	0.79	<0.001	0.11	0.76	0.07	0.82	<0.001	0.13		<0.001	0.90	0.90
	N	1504	1453	1504	1500	1504	1504	1453	1504	1500	1504	1503	1502	1504
TPO	R	-0.04	-0.03	-0.14	-0.07	-0.03	-0.03	-0.02	-0.13	-0.05	0.29	1.00	-0.02	-0.06
antibodies	p	0.14	0.23	<0.001	0.01	0.20	0.23	0.51	<0.001	0.06	<0.001		0.41	0.03
(IU/mL)	N	1504	1453	1504	1500	1504	1504	1453	1504	1500	1503	1504	1502	1504

Urine	R	-0.02	0.00	-0.16	-0.01	-0.04	0.00	0.02	-0.16	0.03	0.00	-0.02	1.00	0.12
collection	p	0.38	0.96	<0.001	0.74	0.10	0.93	0.48	<0.001	0.32	0.90	0.41		<0.001
time (weeks)	N	1503	1452	1503	1499	1503	1503	1452	1503	1499	1502	1502	1503	1503
Serum	R	-0.02	-0.04	-0.01	0.00	0.00	-0.03	-0.04	-0.01	0.00	0.00	-0.06	0.12	1.00
collection	p	0.34	0.16	0.83	0.86	0.85	0.22	0.15	0.77	0.90	0.90	0.03	<0.001	
time (weeks)	N	1505	1454	1505	1501	1505	1505	1454	1505	1501	1504	1504	1503	1505

Abbreviations: N, number of subjects; p, p-value; R, Spearman correlation coefficients, TG, thyroglobulin; TPO, thyroperoxidase

[a] Only includes subjects with urinary creatinine concentrations between the 10th and 90th percentiles (i.e., 41-233 mg/dL). Results were similar in analyses involving all creatinine concentrations (data not shown)

[b] Creatinine adjusted analyte concentrations using residuals method.

Table S2. Maternal thyroid hormone concentrations by increasing quartiles of urine perchlorate and other analyte concentrations[a]

		T4 (μg/dL)	fT4 (ng/dL)	TSH (μU/mL)
	N	Mean[b] (95% CI)	Mean[b] (95% CI)	Mean[b] (95% CI)
Perchlorate (μg/L)				
<4.5	365	12.58 (12.38, 12.79)	0.89 (0.86, 0.91)	1.31 (1.21, 1.40)
4.5-6.8	370	12.29 (12.09, 12.48)	0.86 (0.84, 0.88)	1.37 (1.29, 1.46
6.9-10.1	368	12.24 (12.04, 12.43)	0.84 (0.82, 0.87)	1.36 (1.27, 1.45)
>10.1	373	12.05 (11.85-12.25)	0.84 (0.82, 0.86)	1.42 (1.33, 1.51)
Trend p-value[c]		0.008	0.005	0.15
Iodide (μg/L)				
<90.6	354	12.36 (12.15, 12.57)	0.87 (0.84, 0.89)	1.30 (1.21, 1.40)
90.6-160.0	352	12.29 (12.09, 12.49)	0.85 (0.83, 0.87)	1.39 (1.30, 1.48)
160.1-277.0	359	12.37 (12.17, 12.57)	0.86 (0.84, 0.88)	1.37 (1.28, 1.46)
>277.0	360	12.21 (12.01, 12.41)	0.86 (0.84, 0.88)	1.40 (1.31, 1.49)
Trend p-value[c]		0.44	0.86	0.26
Thiocyanate (μg/L)				
<519	360	12.61 (12.41, 12.82)	0.86 (0.84, 0.88)	1.25 (1.15, 1.34
519-932	371	12.31 (12.12, 12.51)	0.87 (0.85, 0.89)	1.41 (1.32, 1.50
933-1620	370	12.18 (11.99, 12.38)	0.85 (0.83, 0.87)	1.33 (1.24, 1.42)
>1620	375	12.05 (11.85, 12.26)	0.86 (0.83, 0.88)	1.46 (1.37, 1.56)
Trend p-value[c]		<0.001	0.59	0.01
Nitrate (mg/L)				
<36.3	366	12.50 (12.28, 12.72)	0.87 (0.84, 0.89)	1.42 (1.32, 1.52)
36.3-55.5	370	12.29 (12.09, 12.48)	0.84 (0.82, 0.86)	1.38 (1.29, 1.47)
55.6-81.9	367	12.20 (12.00, 12.40)	0.88 (0.86, 0.90)	1.36 (1.27, 1.45)
>81.9	369	12.16 (11.95, 12.36)	0.85 (0.83, 0.87)	1.31 (1.21, 1.40)
Trend p-value[c]		0.04	0.75	0.13

Abbreviations: CI, confidence interval; fT4, free thyroxine; N, number of subjects; T4, total thyroxine; TSH, thyroid stimulating hormone

[a] Only includes subjects with urinary creatinine concentrations between the 10th and 90th percentiles in all subjects (i.e., 41-233 mg/dL)

[b] Adjusted for urinary creatinine, urinary thiocyanate, maternal age, maternal education, ethnicity (Hispanics vs. non-Hispanics), and gestational age at serum collection

[c] Trend test p-values to assess linear dose-response relations were derived by using proc GLM to model an ordinal variable representing quartiles of exposure to perchlorate, iodide, thiocyanate, or nitrate, with each participant assigned the mean value of their respective quartile
